# Supplementary material for: Cholera past and future in Nigeria: Are the Global Task Force on Cholera Control’s 2030 targets achievable?
Source: PLoS Negl Trop Dis. 2023 May 1;17(5):e0011312. doi: 10.1371/journal.pntd.0011312 (PMC10174485; doi:10.1371/journal.pntd.0011312)
Supplement: S1 Information — The analysis includes R calculations, variable importance and model fitting for the full dataset. (DOCX) [file pntd.0011312.s001.docx]

**S1 Information: Sensitivity analysis using confirmed and suspected cholera cases.** The analysis includes R calculations, variable importance and model fitting for the full dataset.

The data for the confirmed and suspected cholera cases had R calculated for 16 states (compared to 6 in the original model), which met the >40 cases thresholds for inclusion. The new dataset consisted of 5,627 data-points for variable importance and model fitting (compared to 279 in the original model). The variable importance plot (shown below) was similar to the variable importance for the original model, with only minimal changes in covariate importance order. This suggested that only small changes would be found in terms of the best fit model to the new dataset.

The new model did not improve model fit in terms of predictive power (shown below) and the same best fit model was selected. Any changes in the performance metrics were negligible (0.001 difference) and there was a slight decrease in correlation (0.71 in the new model), potentially due to the larger dataset creating greater variation. The sensitivity analysis using all the data, proved that the original model was robust, and that the smaller dataset did not bias the results.

**The variable importance for the twenty-two covariates considered for model inclusion.** A serial interval of 5 days (with 8 days SD) was used and the numbers represent the clusters. SPEI01, 12, 48 - Standardised Precipitation Index calculated on 1, 12 and 48 month scale. PDSI - Palmers Drought Severity Index. MPI - Multidimensional Poverty Index. OCV – Oral cholera vaccination.
