# Supplementary figures and images for: Cholera past and future in Nigeria: Are the Global Task Force on Cholera Control’s 2030 targets achievable?
Source: PLoS Negl Trop Dis. 2023 May 1;17(5):e0011312. doi: 10.1371/journal.pntd.0011312 (PMC10174485; doi:10.1371/journal.pntd.0011312)

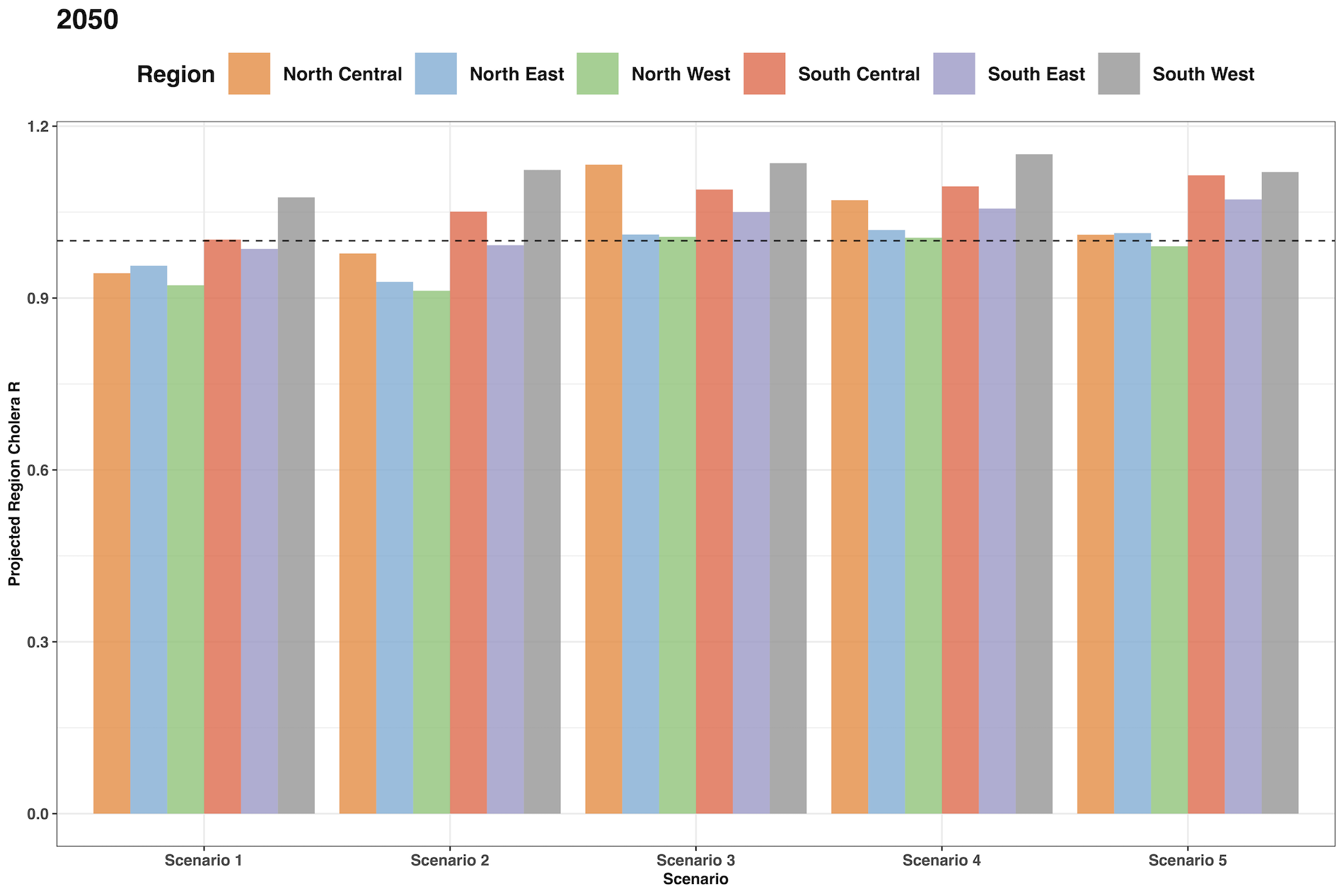

Supplement: S1 Fig — Average regional R value for each scenario at 2050. The regions are based on the six Nigerian geopolitical zones. North Central: Benue, Kogi, Kwara, Nasarawa, Niger, Plateau, Federal Capital Territory. North East: Adamawa, Bauchi, Borno, Gombe, Taraba, Yobe. North West: Jigawa, Kaduna, Kano, Katsina, Kebbi, Sokoto, Zamfara. South East: Abia, Anambra, Ebonyi, Enugu, Imo. South Central: Akwa Ibom, Bayelsa, Cross River, Delta, Edo, Rivers. South West: Ekiti, Lagos, Ogun, Ondo, Osun, Oyo. (TIFF) [file pntd.0011312.s002.tiff]

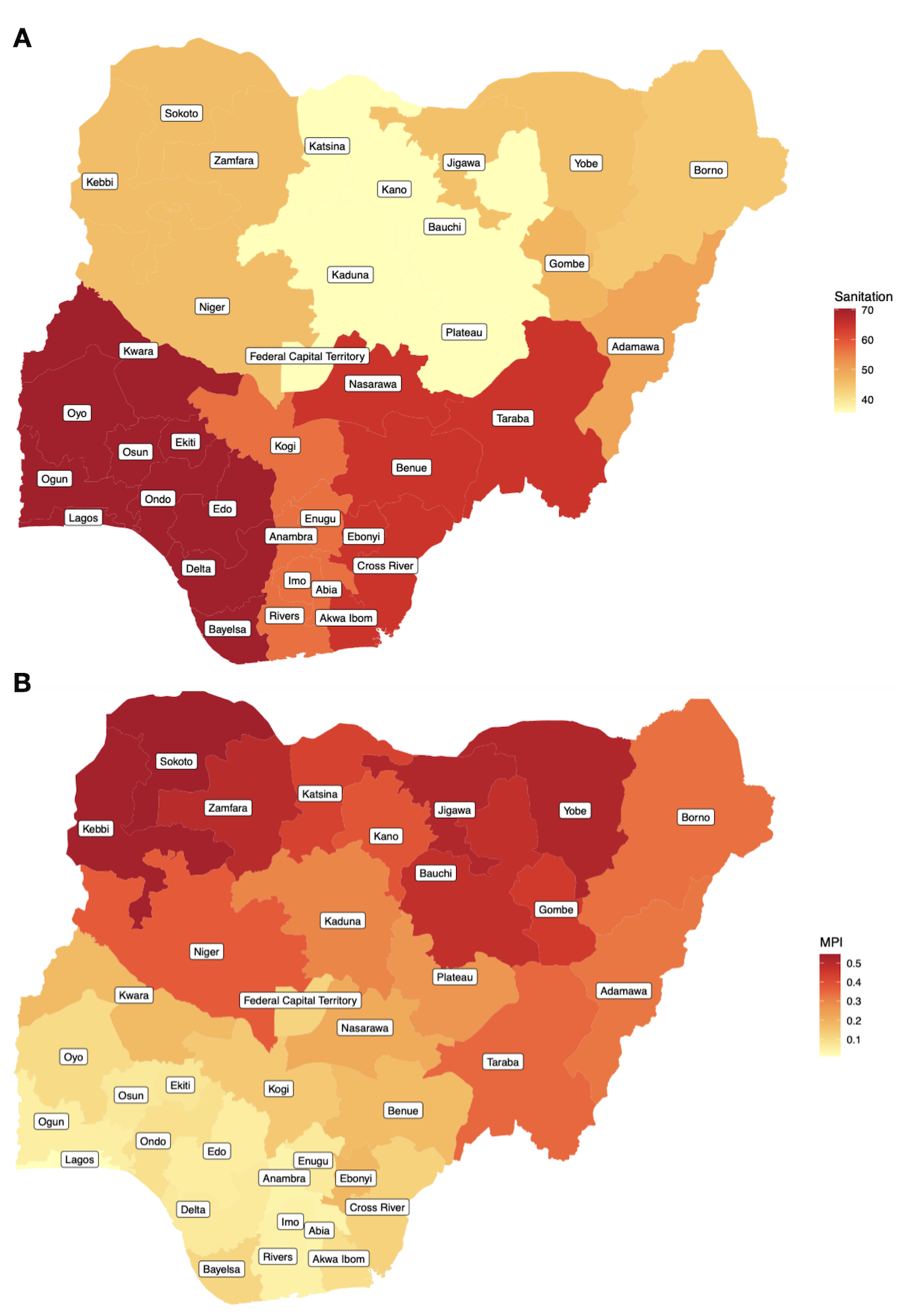

Supplement: S2 Fig — Average values for the full dataset by state, for A, percentage access to sanitation and B, Multidimensional Poverty Index (MPI). The sources and timescales of the data are shown in Table 1. License: CC-BY, available from: https://data.humdata.org/dataset/cod-ab-nga. (TIFF) [file pntd.0011312.s003.tiff]
