# Supplementary material for: Cholera past and future in Nigeria: Are the Global Task Force on Cholera Control’s 2030 targets achievable?
Source: PLoS Negl Trop Dis. 2023 May 1;17(5):e0011312. doi: 10.1371/journal.pntd.0011312 (PMC10174485; doi:10.1371/journal.pntd.0011312)
Supplement: Attachment — Submitted filename: Reviewer Response 1.pdf [file pntd.0011312.s004.pdf]

Response to Reviewer Comments - Cholera past and future in Nigeria: are the Global Task Force on Cholera Control's 2030 targets achievable?

We thank all three Reviewers for their detailed review of our manuscript and carful responses to help improve the research presented here.

The main aim of our manuscript was to use a modelling approach to understand the achievability of the GTFCC targets in Nigeria. The novel aspects of this work are the projections and the discussion and we agree with some of the reviewer feedback that the main messages of the manuscript may have been lost in the extensive methods section, which had three components (the historical analysis, the generalised linear model (GLM) and the random forest model).

To help focus our manuscript, we have simplified our methods, using one model and expanded on the description of the model used, which was the random forest model, with the removal of the GLM and historical analysis. Changing our methods to use one methodology only marginally changed our results, and provided more focused evidence and limitations for our discussion.

By using one methodology, we subsequently used one dataset, which was the best dataset we had available and arguably the best data for cholera in Nigeria. We feel that our approach helped to address some of the concerns raised regarding data limitations.

Through the extensive changes to the methods and restructuring of the manuscript we believe this has improved the readability of the paper and interpretation of our results.

Methods

Reviewer #1:

This is an exploratory study, and not testing any hypothesis. Although, study design is not a problem, there are several methodological issues as given below:

1. The study used the death rates in the analysis. Death is mostly depended on the health system of the country that includes health infrastructure and communication system. Therefore, it could describe the status of the health system of the place, but I do not think analyzing the deaths could predict cholera situation in a country.
2. Cholera death data were taken from two open access sources. It is not clear how the data from the two sources were reconciled. Also, the study used the data until 2016. Since cholera situation changes over time, not including the most recent data set (2017-2022) may yield incorrect forecasting.

> *The historical analysis section of our results has been removed and instead our results focus on the random forest model used to project cholera and how this addresses our study aim of understand the achievability of the GTFCC targets.*

3. The study used the projected climatic data of 2050 and 2070 from WorldClim. There could be several human interventions in future that could change the climatic condition of an area.

> *We agree with this statement and suggest the limitations of all projections in the Discussion (lines 451-460). We agree that human interventions will be highly influential and the discussion section largely focusses on this.*

4. The definition of the cholera outbreak occurrence is not clear. Do they define it by at least one reported case?

> *We have chosen to focus on the random forest models, which used cholera reproduction number as the outcome variable, which has been explained in the methods.*

5. There is no clarity in the method section about the unit of analysis and how many units were there to analyze the data. Different environmental and social data were collected at different geographic scales. How the data at different geographic scales were processed for fitting in the model?

Table 1 provides additional information on data granularity

6. As I see the covariates were selected using a bivariate (should NOT call call univariate) regression model where the covariates were found to be significantly associated with the outcome variable at a 10% confidence limit. Next, the authors said, "The best fit model was identified based on Bayesian Information Criterion and area under the receiver operator characteristic curve." It is not clear how the different models were created from which the best model was derived?

By focussing on one model, the random forest model, we have expanded our methods to include more specific detail, including how the best fit model was ascertained.

7. I don't see validation of the forecasting model. Without validating the forecasting model, how one could forecast cholera situation in 50 years from now.

The ARIMA forecasting has now been removed. In terms of the cholera projections, forecast is perhaps not the correct term here, and has been changed in the instance it was used in the introduction. The methods here do not aim to ascertain what cholera will be doing in 50 years. Instead, we use a relatively simple model to make cholera projections, based on available data and then state, based on these relationships, the potential projected cholera risk under different conditions. The limitations of doing so are in the limitations section of the discussion (lines 451-460).

8. In Figure S4, it is said that most of the correlations between the covariate and the WHO cholera deaths are significant, potentially due to a lack of complete data. What is meant by lack of complete data. When the data is incomplete what will be the implication of the findings of this study? Also, how do the authors know that the cholera death data are underreported. I am also wondering why a national average was taken from the available data points and used for all years (and not the average of year before and year after) when they found missing data of a year. The method used for interpolating the data would dilute the year wise variation in the data.

Figure S4 has now been removed, as it related to the methods from the original submission. In terms of data completeness, how incomplete data was dealt with in the model (random forest) is in the methods section (which now provides more specific detail of the model), and limitations of the data are in the limitations section of the discussion.

9. It is said that five projection scenarios were created to project cholera to 2070 using the two models. Which specific model was used for which scenario?

One model (random forest model) is now used.

Reviewer #2:
The main points of the methods get lost in the detail. There are a lot of different models noted in the methods section, but in the introduction it indicates that only two different models were used. It is unclear what the two different models are. Is it a GLM and random forest models? Or is it two of the same model type with different outcome measures? Is it national and sub-national? What is the purpose of the two different model types in answering the research question? There needs to be a more explicit explanation of what models were used (potentially visualized in a table or figure), the variables included in each model, and how each model is used to answer the research question.

We agree that the main points were lost in the methods section of the previous manuscript, and as stated, we have now simplified the methods to include one model.

Some other specific recommendations:

94 – More comparable to what? Other countries? To compare death rates across different regions of Nigeria?

96 – “Cholera death data were taken from two open access sources.” is a full sentence. There should be a period at the end not a comma.
163-164 – This should be split into two sentences.

As the historical analysis was removed, to help focus the methods, the death rates are no longer in the manuscript.

182-196 – Which SDG’s is this referring to? All of them? Just the ones related to climate?

The specific SDGs used in the scenarios are described in the Scenarios and Projections section of the Methods, none of the SDGs were used in the PDSI projections (lines 197-207), which instead used RCP projections.

206 – Is there research to support this assumption? This seems like a significant assumption to make. Water withdrawl does not indicate who is using the water (or that water access is increasing for everyone). It may mean more water collected by specific subsets of the population but not for others. Water withdrawl also does not specify what the water is used for. More water may be withdrawn during a drought scenario to support increased need for agriculture, not necessarily consumption.
223-224 – This is not a full sentence.
258-259 – It would be helpful to highlight the differences in the data collection methods that may explain why the data from these two sources is so different. Were they surveying similar study populations? Where they looking at different indicators of cholera mortality (laboratory confirmed vs. reported)?, etc.

Water withdrawal was selected in the GLM and not the random forest model, therefore the discussion regarding water withdrawal is now removed. We have used one data source for each covariate in the random forest model (Table 1), which reduces the difficulties in comparing the limitations of different dataset.

Results
Reviewer #1:
The results are presented according to the analysis plan. However, since there are problems in the data, I do not think the findings of the study have any implication in real life.

The only cholera data now used is the data fit to the random forest model, which is detailed surveillance data provided by the Nigeria Centre for Disease Control (NCDC). The data is the best available data for cholera in Nigeria, nevertheless we discuss the limitations of this and all cholera data in the Limitations section of the Discussion (lines 443-449) and Axis 1 in the Update on the Roadmap and 2030 Targets section.

Reviewer #2:
Some minor changes to tables and figures may help with the clarity of the results:
Fig 4 – The unit on the y axis should be explained better. In the methods it indicates that outbreak occurrence of 1 indicates at least one cholera case but the interpretation of values lower than 1 should be explained.

This figure has now changed to represent the new methods. Cholera R is described in the methods (lines 129-141).

Figure 5 – The color scale on this is confusing. The cut toff point of 1 is important for for the purposes of these results, but it is difficult to tell specific values with a continuous color scale. Would a categorical color scale showing <1,1 (+/-0.1),>1 be possible to more clearly visualize reductions and improvements?

We agree that R greater or less than 1 is important for these results, but by making the cholera scale binary, this would lose some of the complexities of the relationship. Additionally, R less than or

greater than one is generally an indication, not certainty, of increased/decreasing cases, therefore to set an absolute threshold at zero may make the results misleading for certain states (particularly those close to 0). An additional figure in the Supplement (Sup Fig 1) helps identify which states/ regions were over or less than 1 by mid century.

Conclusions

Reviewer #1:

The conclusions are supported by the results and limitations of the study are briefly described. However, I am afraid the findings of the study have any public health relevance.

Thank you for your feedback regarding the public health relevance of our study. Here we use a modelling exercise to inform cholera policy in Nigeria and describe how our results fit into global cholera targets. We therefore argue that our results have comparatively more public health relevance by attempting to understand how our results can inform policy and therefore impact peoples' health.

Reviewer #2:

Many of the points in the discussion and conclusion are limited by either lack of support by the results presented in this analysis or by previous literature:

346-354 – It needs to be stated more explicitly here why sanitation access is a concern for cholera transmission. Explain how limited sanitation access increases risk of cholera transmission (then later why this justifies improvements in environmental sampling). The correlation analysis alone also cannot prove that limited sanitation is the reason for cholera outbreaks because the analysis does not account for potential confounding by other variables (i.e. poverty). The language here needs to be careful to not draw causal conclusions outside of the scope of the analysis.

The correlation matrix referred to here has now been removed and replaced by one which supports the covariate selection process (Fig. 3). We agree strongly that poverty is important in sanitation and the Discussion focusses heavily on both of these social factors. The model accounts for both poverty and sanitation and we have also included an additional few sentences in the Discussion about sanitation, poverty and transmission (lines 293-297).

361-363 – Is this saying that reducing the size of rural populations would reduce cholera outbreaks. What about the increased risk of cholera transmission in more dense urban settings? What is meant by "effective urban planning"? Is this just in terms of WASH access or is this also referring to increased climate resiliency, etc.?

The paragraph here is suggesting reasons why the north of the country generally has a higher cholera burden, e.g. a more rural north has resulted in less development. We agree that densely populated informal settlements are a risk factor for cholera and an additional sentence and references have been added on this, we also expanded on the meaning of "effective urban planning", in this instance (lines 305-216).

374 – It's unclear what "and via introductions" means.

This refers to the introduction of new cases and strains from international travel, and has now been stated more clearly (lines 327-330).

376-382 – The conclusions about sub-national cholera projections are all be based on S1 and are ignoring the other scenarios. The broad conclusion that southern states will do better and northern states will do worse does not hold true for S3-S5. It seems like a big stretch to conclude that the sub-national projections are more optimistic when all scenarios show variation across different regions (with some areas having increases in cholera and some having decreases based on the scenario).

The conclusions regarding the projections, in this case the sub-national projections, state that according to our results, all R values were less than one by 2030 in the southern states under S1. However, it also states that in worsening conditions, the north of the country doesn't project a

significantly worse cholera risk, whereas, the southern states do. All of this is stated in the Discussion, the optimism refers to the fact that there is significant spatial heterogeneity in the results that is not captured from the national projections, as some states are perhaps much closer to the 2030 targets than others.

379-381 – This statement doesn't align with what is shown in Figure 5. Northern states showed an initial reduction in 2050 but then a slight increase in 2070. Based on what is shown in the figure, more time would not lead to a reduction in transmission.

Some areas and scenarios do see an increase from 2050 to 2070, which is now discussed in the Results sections, for the new national scenarios (lines 251-257). Whereas, some areas do not see increasing R values, showing the significant heterogeneity across the country.

The increase at 2070 is due to changing environmental conditions (2050 and 2070 were the projected values available), which is potentially due to the methods used. The drought incidences used here had very high variable importance based on node impurity (see new Fig 3) and therefore only small changes in the projected PDSI, resulted in changes in the projections. For example, the increase in some states in S3, from 2050 to 2070, is due to PDSI projections being closer to 0 in 2050 and then decreasing in 2070, as the relationship between PDSI and cholera risk is multi-directional. This has been added to the Results at lines 251-257.

403-422 – It is unclear how the data presented in this analysis supports a need for more testing. While this is an important strategy to pursue to collect better data for the GTFCC roadmap, it is outside of the scope of this analysis. It needs to be more explicitly stated why the results from this analysis lead to this recommendation.

We believe that the previous analysis of the historical data, which had significant discrepancies and missing values, showed the need for improved data collection and testing. As this section has now been removed, how improved testing and surveillance could improve the data used here is now stated in this section (Axis 1 in Update on 2030 Roadmap and Targets).

442-444 – This sentence is conflicting with the previous paragraph which suggests that the GTFCC roadmap should shift from outbreak response to development. Strengthening healthcare would potentially improve outbreak response but not necessarily development. Strengthening healthcare is also an overly broad recommendation. What specifically about healthcare needs to be improved? This, again, seems to fall outside the scope of this analysis. This claim needs to be supported by either the data presented in this analysis or by previous literature.

We agree that strengthening healthcare would improve cholera outbreak response along other health outcomes which are important for development, making it a cost effective method of tackling cholera, vs temporary outbreak response. In this section we make many specific recommendations, including improving human resources by raising the profile of healthcare workers, greater resource and service availability and improvements to public spending, which are all Nigeria specific examples and referenced (lines 393-416).

449-454 – All sentences in this paragraph need references.

The paragraph is correctly referenced, with the reference at the end of any sentences it refers too.

463-464 – Reference? What is this approach and how does it relate to this analysis?

These approaches are referenced in 58-60, and relate to the results that conflict was influential in cholera transmission and included in the best fit model. We believe that the new methods have made this clearer.

In general, it is unclear how the recommendations to the GTFCC axes are specifically expected to achieve the goal if 90% reduction by 2050, and, more importantly, how the evidence from this analysis support the recommendations. The data from this analysis shows that none of the

projections would lead to a 90% reduction in Cholera, even by 2070, so how is it expected that these recommendations would lead to improvements by 2050? The methods used in this analysis are not able to draw conclusions about causality (i.e. what specifically is causing the increases or reductions in cholera). It seems to stretch outside of the scope of the analysis to recommend specific changes given the lack of support from this analysis about what specifically is causing increases or reductions in cholera.

The reasons for the 2050 target is suggested in Discussion, section "Evidence for the Sub-national Projections", the southern states do reach the targets by 2030, according to our projections, and therefore our discussion largely revolves around trying to reach this across the country by addressing regional inequity (lines 333-340). The issue of the 90% target, in terms of this analysis, is stated in the limitations section (lines 462-467).

Editorial and Data Presentation Modifications
Reviewer #2:
Other suggestions for the introduction section:
46 – Targets are not a type of strategy for addressing challenges, they are a set of goals that can be used to identify best strategies for addressing challenges. The language here could be more precise.

Language changed (lines 45-47)

48 – What types of institutions are included? Are these academic? Governmental? NGO?, etc.

Institution types included (lines 48-49)

58 – Language here is confusing. Is "they" referring to the goals? Or the organizations?

Clarified the "they" refers to the goals and targets (lines 59-60)

60 – "Gains in cholera control" is ambiguous. Does this mean the prevalence of cholera has gone down? Or that strategies to reduce cholera have been more widely implemented and/or have been more effective? Does "at the local level" mean in specific regions of Nigeria? Or throughout all of Nigeria? More specificity in what progress has been made would be helpful here.

It has been clarified that this is in reference to the implementation of strategies and by "local", this refers to sub-national, not country level implementation (lines 60-63).

82 – Would be helpful to briefly explain here what the two models are and how they are used to answer the research question.

One model is now used (random forest), and more explanation of how it is being used to answer and understand the study aims is given in the introduction (lines 80-90).

Summary and General Comments
Reviewer #1:
This study attempted to forecast cholera situation in Nigeria for almost 50 years from now using historical data of cholera death from two different sources. I find this an ambitious project as this study used the data of the last 40-50 years when I do not think there was systematic disease surveillance for keeping the records of morbidity and mortality in that country. The nature of data could result in incompleteness, inconsistency, and incorporating falls positivity that could mislead forecasting of the cholera situation in the country. Therefore, I do not think the outcomes of this study have any practical implacability.

See response for Methods comment 7. The study here aims to use a relatively simple model, based on the best available data, to make cholera projections. The study aims to use these relationship to understand how cholera risk may change in the future. In terms of the data used, cholera data are

lacking and several inconsistencies remain globally, but we do not believe that this should prevent or discourage research in this area. Our recommendations and discussion are made with this in mind, including the limitations of our methods and data.

Reviewer #2:
The methods appear to be thorough and the results have potential public health relevance, but there are significant limitations in the writing and interpretation of the results. Several points in the discussion section are not supported by evidence from the analysis or other relevant literature. To be ready for publication, this paper either needs an adjustment to the language and interpretations or additional analyses to support the conclusions about causes of cholera and intervention recommendations.

We have significantly restructured the methods and results section of the manuscript, which we hope has helped with the readability of our paper and the interpretation of the results. We make recommendations based on the covariates selected in the best fit model, and do not aim to make recommendations beyond these, as we have either not studied the relationship or it was not found influential in the methods we used. All recommendations have been referenced and we have given multiple examples, specific to Nigeria, throughout.

Reviewer #3:
The authors have submitted an interesting manuscript questioning the likelihood of Nigeria reaching its 2030 goals regarding cholera control. They have used the WHO Global Health Observatory and the Global Health Data Exchange is their primary sources for cholera mortality and have used WorldClim for climate information. In addition, subnational data were also used to analyze regional differences within Nigeria. By most possible scenarios, including the most likely, the 2030 goals will not be met.

Here are a few comments and questions:

1. Please give more details regarding the data sources, including their methods and potential pitfalls

Table 1 has included more information on data sources and granularity. In terms of data limitations, this is discussed in the Limitations and Axis 1 in the Discussion (lines 361-390 and 443-449).

2. It was not clear what data have been used to estimate its impact on cholera mortality, including how much of the impact was directly related to available potable water.

See above comments, some of the data that is being referred here is likely removed due to the new simplified methods.

3. What are the confounders for the use of data in a linear manner, especially as it relates to accuracy of data acquisition.

The generalised linear model has now been removed and therefore we are no longer assuming linear relationships in our data or outcome variable.

4. Also, mortality may change in the setting of the same case load if access to or quality of health care changes.
5. It appears that the GHDx data begin in 1990, right where there is a major peak in the WHO data. First, what is the impact for the trend if the GHDx data begin at the peak, which is the highest rate for any of the WHO data? Second, do you have any way to reconcile the differences in data between the two data sources.

Data sources removed with the historical analysis. The only cholera data now used are surveillance data from NCDC.

The potential role of immunization should be addressed to a greater extent since there is good evidence from Bangladesh that there is herd protection from immunization that could affect the incidence of cases (see Chowdhury, CMR, 2022).

> *Immunisation was addressed in the methods used here using OCV data provided by NCDC, this was transformed to a binary outcome variable and included in the covariate selection (lines 105-109). It was not included in the best fit model and therefore was not a focus of the discussion here.*

There are also numerous small grammatical issues that must be corrected. A few are shown below:
Line 242 – Do the authors mean defecation rather than defection?

> *Typo corrected*

Lines 261, 385, 473 – These sentences begin with a conditional conjunction (whereas, while) that requires the conditional component. As written, they are incomplete sentences.

> *Line 261 is now removed with the previous methods. Line 385 and line 473 corrected.*
